# Burden of neural tube defects in India: a systematic review and meta-analysis

**DOI:** 10.1007/s00381-024-06627-x

**Published:** 2024-10-04

**Authors:** Anuvi Sinha, Ponmani P, Hirok Chakraborty, Rajan Kumar Barnwal, Ratnesh Sinha

**Affiliations:** 1 Obstetrics and Gynecology Department, Sadar Hospital, Ranchi, Jharkhand India; 2 Department of Pharmacology, Karpagam Faculty of Medical Sciences and Research, Coimbatore, Tamil Nadu India; 3 Department of Physiology, Manipal Tata Medical College, Manipal Academy of Higher Education, Manipal, India; 4 Department of Community Medicine, Medini Rai Medical College and Hospital, Palamu, Jharkhand India; 5 Department of Community Medicine, Manipal Tata Medical College, Manipal Academy of Higher Education, Manipal, India

**Keywords:** Neural tube defect, Congenital defects, Birth defects, India, Meta-analysis

## Abstract

**Background:**

One of the most common and serious congenital defects is neural tube defect (NTD) in India. The data about the NTDs in India is lacking. The objective of this meta-analysis is to provide an estimate of NTDs in India with regional variations.

**Method:**

This study was conducted by doing a literature search using PubMed (Medline) and Embase databases for studies published from their inception to 1 October 2023 by using relevant keywords. We have prepared our study protocol by following the Preferred Reporting Items for Systematic Reviews and Meta-Analyses (PRISMA) checklist, and our study is registered in PROSPERO. Pooled prevalence was calculated by using the Der Simonian-Liard method and random effect model to find out the burden of NTD in India. Additionally, subgroup and sensitivity analyses were also performed. NHLBI (National Heart, Lung, and Blood Institute) tool was used for assessing the study quality.

**Results:**

A total of 1129 articles were identified by using the predefined keywords in which 27 articles were selected which were fitting the selection criteria defined in our study. The prevalence of NTDs in our meta-analysis was found to be 9.46 per 1000 births with a 95% confidence interval of 8.01 to 10.91 per 1000 births with significant heterogeneity with I2 of 99.15%.

**Conclusion:**

Our study highlights the increasing trend of NTDs over the past decades, with significant regional variation in India. There is an urgent need for comprehensive prevention strategies such as advocacy and awareness, antenatal screening for NTDs, folic acid supplementation, and food fortification. Future research is required for identification and implementation for a target-based approach for region specific.

## Introduction

Neural tube defects (NTD) are deformities of the central nervous system (CNS) that occur during fetal embryonic development due to abnormal neurulation process and incomplete closure of the neural tube [1, 2]. It presents with a wide spectrum of clinical features including anencephaly, spina bifida, encephalocele, myelomeningocele, and others [2, 3].

NTD is the second most common type of congenital defect after congenital heart defects. Its etiology is multifactorial, a composite of genetic and environmental causes, of which the most common is folic acid deficiency during the first trimester of pregnancy. The chromosomal anomalies associated with NTD are trisomy 13, trisomy 18, and triploidy among which triploidy with spina bifida being the most common NTD associated with genetic chromosomal abnormality [2, 3].

## Global and national scenario

As per a 10-year prospective observational study done in India, the burden of NTDs is 3.9 per 1000 live births [4]. However, there are geographical variations within the country, with incidence varying from 7.48 per 1000 live births in northern India compared to 3.6 per 1000 live births in South India which may be attributed to dietary, genetic, and health infrastructure differences [2, 5]. Contrary, the incidence of NTD in the United States is 1 per 1000 live births which is 3 times lower than that observed in India and 3 to 5 times lower than in Northern China [6].

The consequences of NTD include live births, stillbirths, and second-trimester abortions. Studies have reported live birth and stillbirth prevalence were 1.3 and 1.7 per 1000 births, respectively, among NTD cases [7].

Indian Government is putting its best effort into providing iron–folic acid supplementation to women, during pregnancy through public health facilities [8, 9]. However, this alone is not enough to prevent the uprise of NTDs. We need reforms in the existing government policies such as vigilant ultrasound surveillance during pregnancy, provision of fortified folic acid food in women of reproductive age group, identification of women who are at increased risk of developing NTD in future pregnancy like a previous history of NTD, and diabetes in previous pregnancy on antiepileptic drugs and provide them with folic acid supplementation in higher doses.

Individual studies are often limited by confounding factors such as sample size, diagnostic tools, and study methodology. Thus, the current systematic review and meta-analysis (SRMA) aims to generate evidence about the burden of NTD in India with geographic variation. The results of our study can be used to strengthen existing government policies and identify the high-burden areas to address this huge silent stumbling block in maternal and childcare.

## Methodology

The current SRMA was performed according to the Preferred Reporting Items for Systematic Reviews and Meta-Analysis (PRISMA) [10] and registered in PROSPERO (Registration id: CRD 42023473350).

### Search strategy and selection criteria

We have searched PubMed (Medline) and Embase for all the relevant articles that were published since the time of their inception till 1 October 2023 in the databases mentioned above by using keywords such as Bifida Spina, Cleft Spine, Dysraphia Spinal, Dysraphism Spinal, Open Spine, Schistorrhachis, Spina Bifida, Spinal Dysraphia, Spinal Dysraphism, Spine Cleft, Spine Open, neural tube defects, anencephaly, encephalocele, and craniorachischisis. After this, it was combined with other keywords to find studies done in India and reporting the burden of NTDs. Finally, we have applied filter for the free-full text and English language.

### Study selection

The articles found in both databases were downloaded and imported into Zotero software for merging and removing duplicate entries. After that, a screening process was initiated, in which we did the title and abstract screening by following the selection criteria by two authors. Articles were assessed and selected for full-text screening based on their relevance to the research question. If there was any confusion about any study, it was selected for full-text screening, and if it persisted, other authors were consulted to reach a common consensus.

## Selection criteria for title and abstract screening

The inclusion criteria for the selection studies were as follows: (1) Observational, Prospective, Cross-sectional, Comparative, Hospital Based, Community-Based, or Prevalence studies and (2) studies reporting NTDs in Indian populations.

The exclusion criteria were as follows: (1) Studies that were Case Series/Reports, Interventional, Meta-Analysis, or Laboratory studies; (2) studies done on non-human populations or outside India; and (3) studies reporting other congenital defects other than NTDs.

In full-text screening, studies with clear information about the outcomes were selected for data extraction and analysis. Those studies which did not have clear or complete information like methodology, outcomes, and results were excluded.

Shortlisted study data were extracted into data summary tables in the following format which included author, publication year, study design, study location, sample size (total number of participants), no of NTDs reported, diagnosis done by which method, and study population.

### Study quality assessment (risk of bias assessment)

NHLBI (National Heart, Lung, and Blood Institute) tool was used to assess the quality of the study of the shortlisted article independently by the two authors; any disagreement or discrepancy was settled by discussing it with other authors [11]. This was used to assess the quality of the study methodology and to look for the possibility of any bias in the study such as in its design, methodology, and analysis. No modifications were done in the checklist for the assessment of the risk of bias.

### Statistical analysis

Der Simonian-Lard (DSL) method with a random effect model with a 95% confidence interval (CI) was used for calculating the pooled prevalence in our study to know the burden of NTDs in India. To assess statistical heterogenicity among studies, *I*^2^ statistics was used in the study, and dissymmetry was measured by plotting a funnel plot. To improve the credibility of our results, sensitivity analysis was done along with subgroup analysis based on different regions and states in India. In our study figures, the prevalence is in proportion format; we have converted the prevalence to per thousand births in the “Results” section for easy interpretation for the readers.

### Role of the funding source

There was no funding source for this study.

### Ethical statement

Ethical approval and patient consent were not required for our study, as it is using the data that of previously published literature.

## Results

### Literature search

**Fig. 1:**
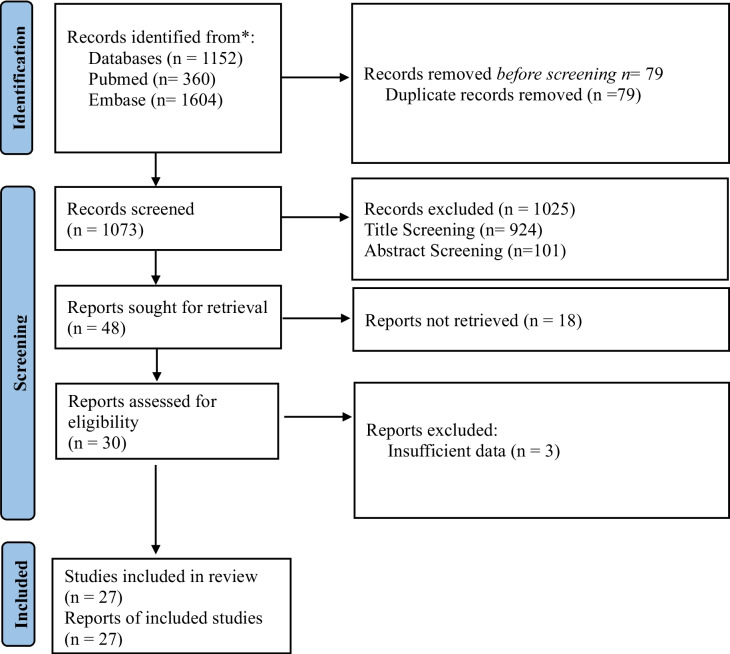
PRISMA flow diagram

By using the keywords defined in our methodology and limiting our search strategy to free full text and English language, our search in PubMed and Embase databases yielded 1152 articles. We removed 79 deduplicates from the combined list of articles and then screened 1073 articles. The complete details of the screening process are shown in the PRISMA flow diagram in Fig. 1. A total of 27 articles were included in the analysis [4, 5, 12–36].

### Primary outcome: prevalence of NTDs

**Fig. 2:**
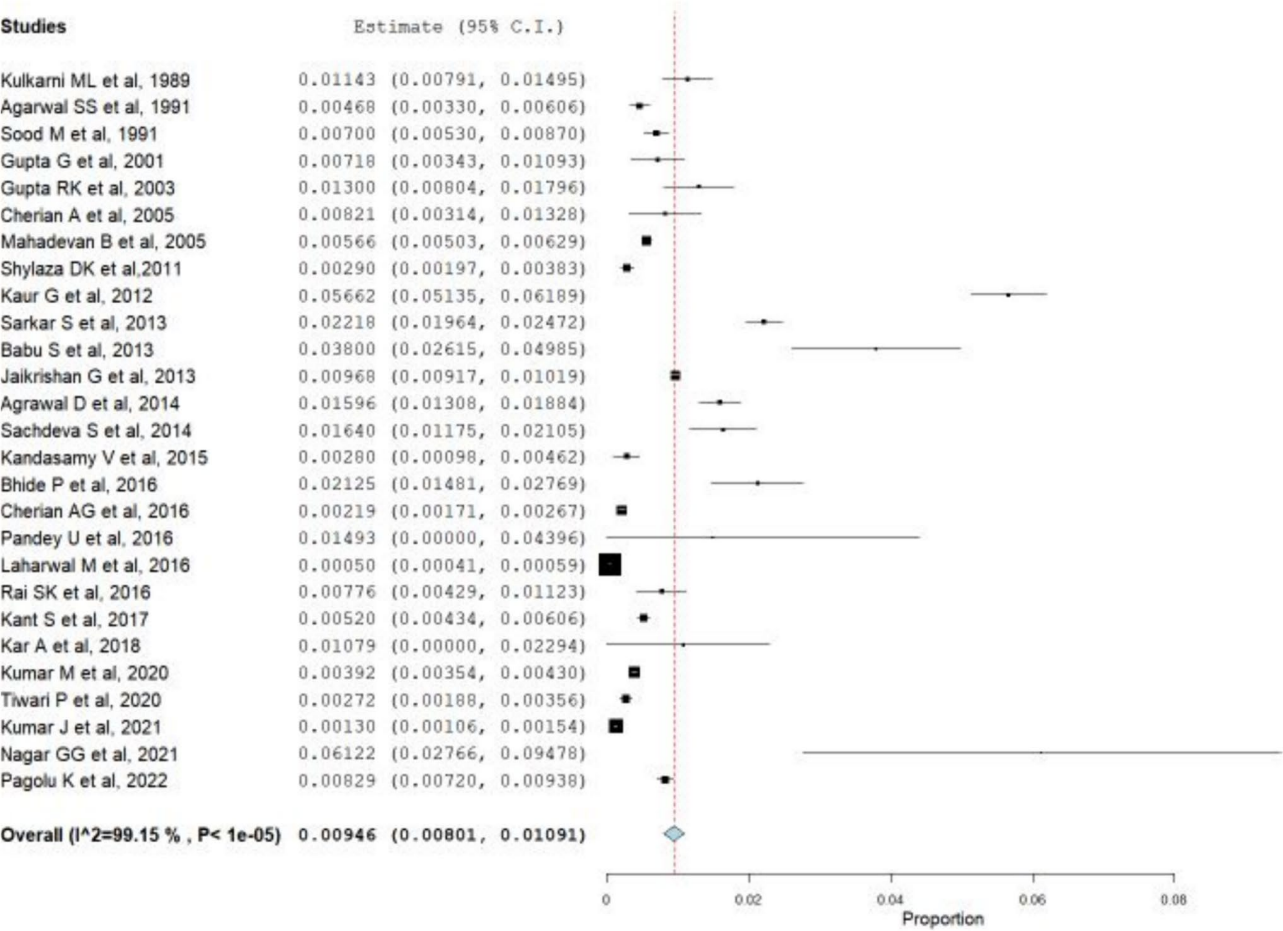
Prevalence meta-analysis of the proportion of neural tube defects in India

Table 1 consists of the summary of the studies included in our meta-analysis. A proportional meta-analysis of the included studies was conducted by following the Der Simonian-Liard method of the binary random effects model. The results revealed a pooled prevalence of NTDs in 9.46 per 1000 births with a 95% confidence interval of 8.01 to 10.91 per 1000 births with significant heterogeneity with *I*^2^ of 99.15%. Figure 2 presents the forest plot of the meta-analysis.

**Table 1:** Summary of the included studies

Study	State	Study type	Diagnosis done	Sample size (*N*)	NTDs (*n*)	Prevalence per 1000
Kulkarni ML et al. [12]	Karnataka	Prospective	Post-delivery	3500	40	11.43
Agarwal SS et al. [13]	Uttar Pradesh	Prospective	Post-delivery	9405	44	4.68
Sood M et al. [14]	New Delhi	Prospective	Checkup/USG	9286	65	7.00
Gupta G et al. [15]	Maharashtra	Prospective	Post-delivery	1950	14	7.18
Gupta RK et al. [16]	Jammu & Kashmir	Prospective	Post-delivery	2000	26	13.00
Cherian A et al. [17]	Uttar Pradesh	Prospective	Survey	1218	10	8.21
Mahadevan B et al. [18]	Pondicherry	Prospective	Post-delivery	54,738	310	5.66
Shylaza DK et al. [19]	Karnataka	Cross-sectional	Post-delivery	12,753	37	2.90
Kaur G et al. [20]	Chandigarh	Prospective	Lab test	7400	419	56.62
Sarkar S et al. [21]	West Bengal	Prospective	Post-delivery	12,896	286	22.18
Babu S et al. [22]	Andhra Pradesh	Prospective	Checkup/USG	1000	38	38.00
Jaikrishan G et al. [23]	Kerala	Prospective	Post-delivery	141,540	1370	9.68
Agrawal D et al. [24]	Orissa	Prospective	Post-delivery	7268	116	15.96
Sachdeva S et al. [25]	Haryana	Cross-sectional	Post-delivery	2865	47	16.40
Kandasamy V et al. [26]	Tamil Nadu	Prospective	Post-delivery	3220	9	2.80
Bhide P et al. [27]	Maharashtra	Prospective	Post-delivery	1929	41	21.25
Cherian AG et al. [28]	Tamil Nadu	Cross-sectional	Post-delivery	36,074	79	2.19
Pandey U et al. [29]	Uttar Pradesh	Retrospective	Questionnaire/record based	67	1	14.93
Laharwal M et al. [30]	Jammu & Kashmir	Prospective	Checkup/USG	248,509	125	0.50
Rai SK et al. [5]	Uttar Pradesh	Prospective	Survey	2450	19	7.76
Kant S et al. [31]	Uttar Pradesh	Cross-sectional	Survey	26,946	140	5.20
Kar A et al. [32]	Orissa	Prospective	Checkup/USG	278	3	10.79
Kumar M et al. [4]	New Delhi	Prospective	Checkup/USG	102,216	401	3.92
Tiwari P et al. [33]	Not mentioned	Retrospective	Questionnaire/record based	14,681	40	2.72
Kumar J et al. [34]	Chandigarh	Retrospective	Questionnaire/record based	86,850	113	1.30
Nagar GG et al. [35]	Rajasthan	Prospective	Checkup/USG	196	12	61.22
Pagolu K et al. [36]	Andhra Pradesh	Cross-sectional	Checkup/USG	26,423	219	8.29

### Sensitivity analysis and subgroup analysis

**Fig. 3:**
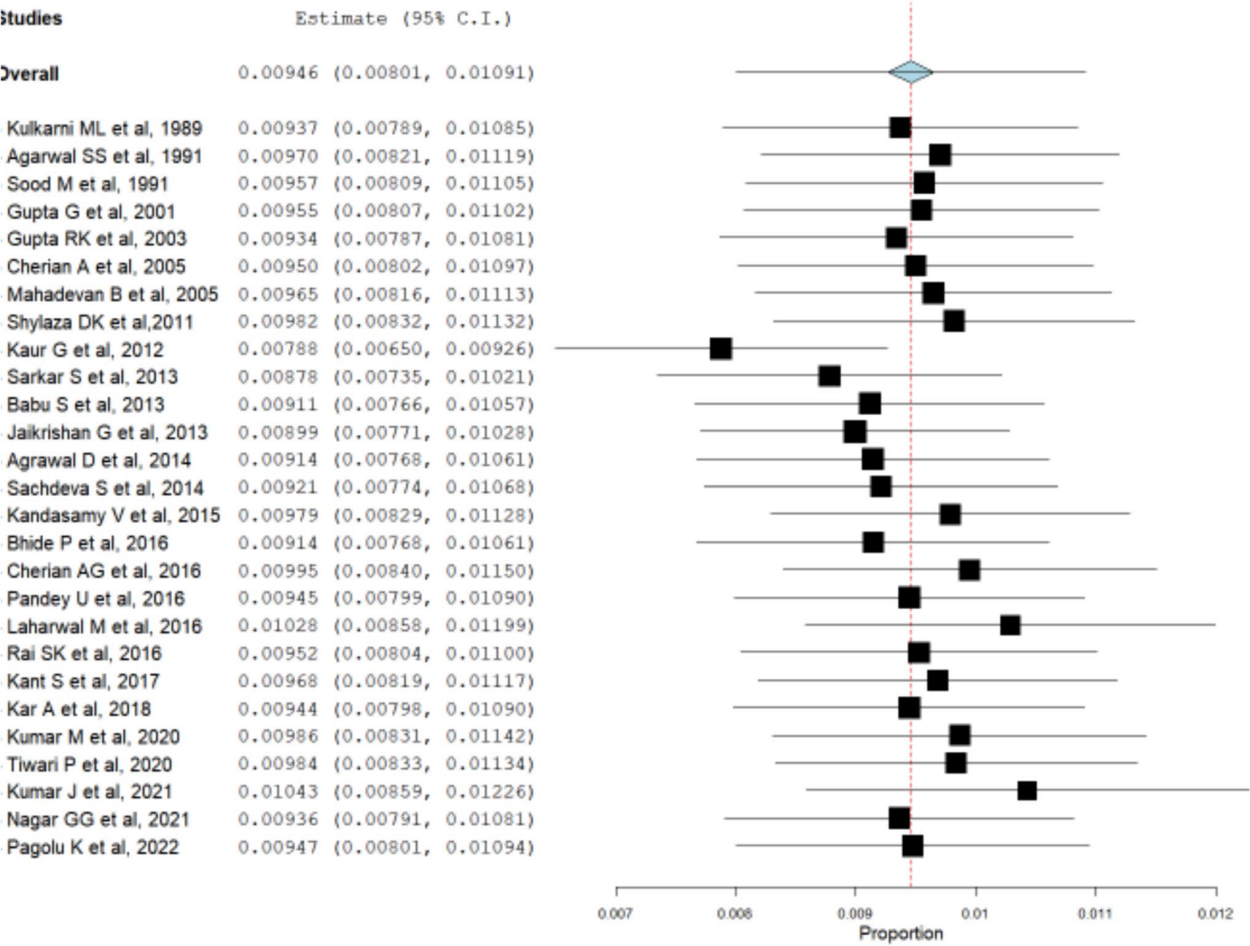
Sensitivity meta-analysis forest plot using the leave-one-out method

Heterogenicity was very high (99.15%) among the included studies; hence, we have performed a sensitivity analysis. To identify the studies that had an overall influence on the effect size, sensitivity analysis by using the leave-one-out method was done. The omission of the study by Kaur et al. [20] seems to have a relatively larger influence, as compared with other studies on the estimation of the overall effect size. The sensitivity meta-analysis is depicted in Fig. 3.

**Fig. 4:**
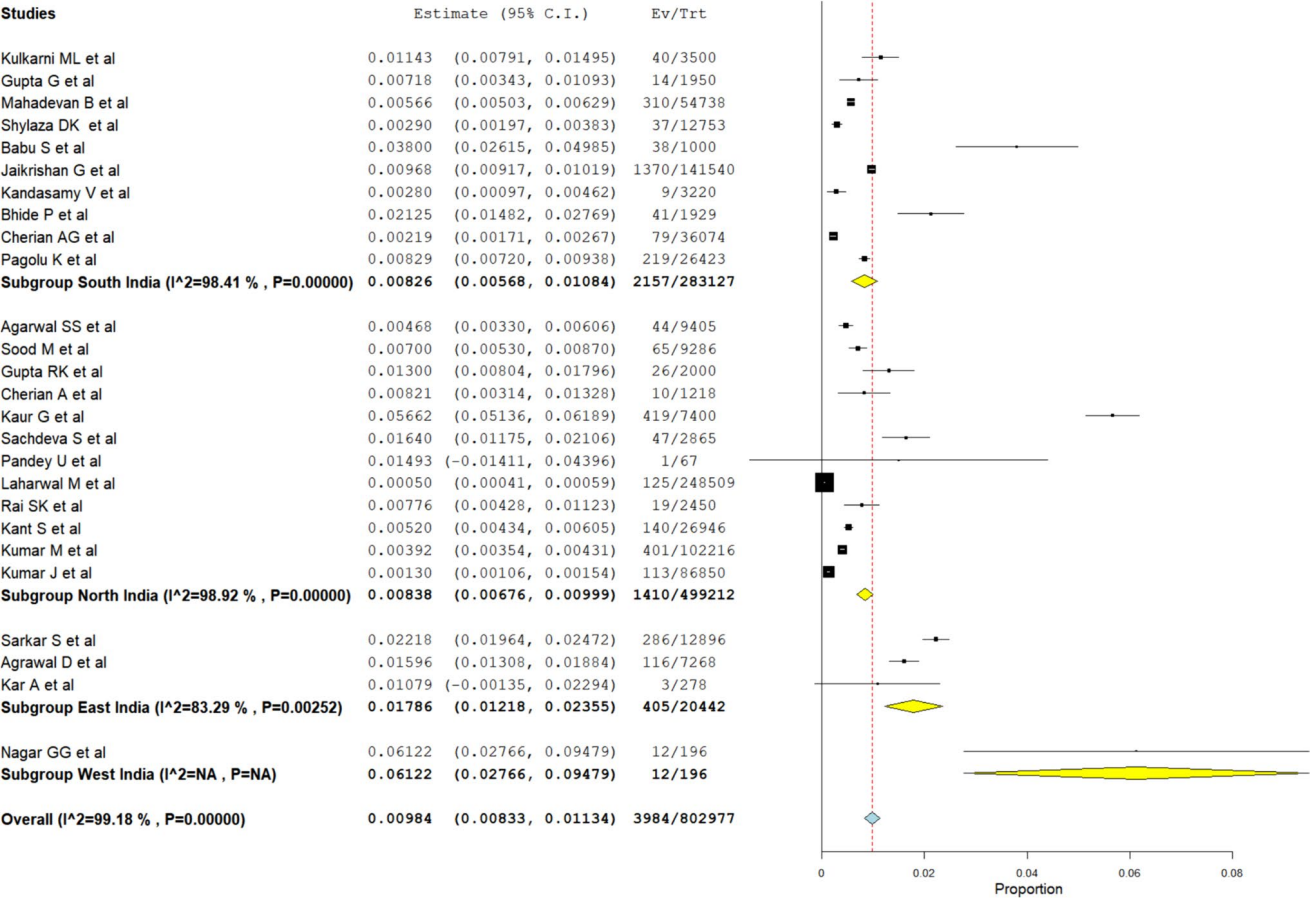
Subgroup meta-analysis forest plot based on different regions of India

A subgroup meta-analysis was conducted to determine the prevalence of NTD in different regions and states of India. The prevalence in the western region of India (Rajasthan) was found to be high (6.12%). But the inclusion of only one study in this category casts uncertainty on the high prevalence. This was followed by Eastern India (1.78%), Northern India (0.84%), and Southern India (0.83%). The results of this meta-analysis are depicted in Fig. 4.

**Fig. 5:**
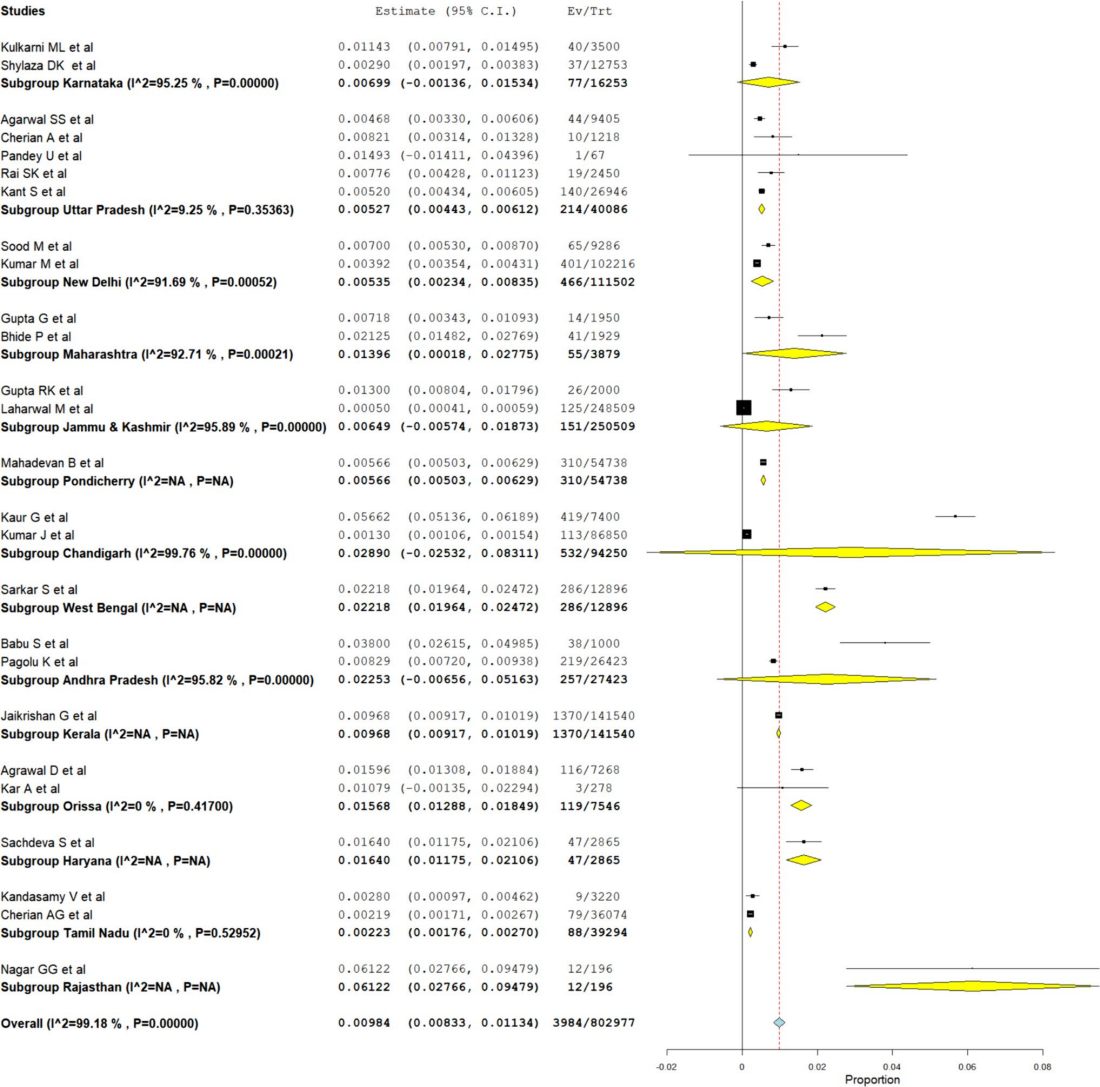
Subgroup meta-analysis forest plot based on different states of India

An additional subgroup analysis was conducted to determine the prevalence of NTD in each state of India (Fig. 5), in which it was observed that the Tamil Nadu state had the lowest prevalence of NTD of around 2.23 per 1000 births with a 95% confidence interval of 1.76 to 2.7 per 1000 births. The highest prevalence of NTD was seen in Rajasthan which was 61.22 per 1000 births ranging from 27.66 to 94.79 per 1000 births.

### Publication bias

**Fig. 6:**
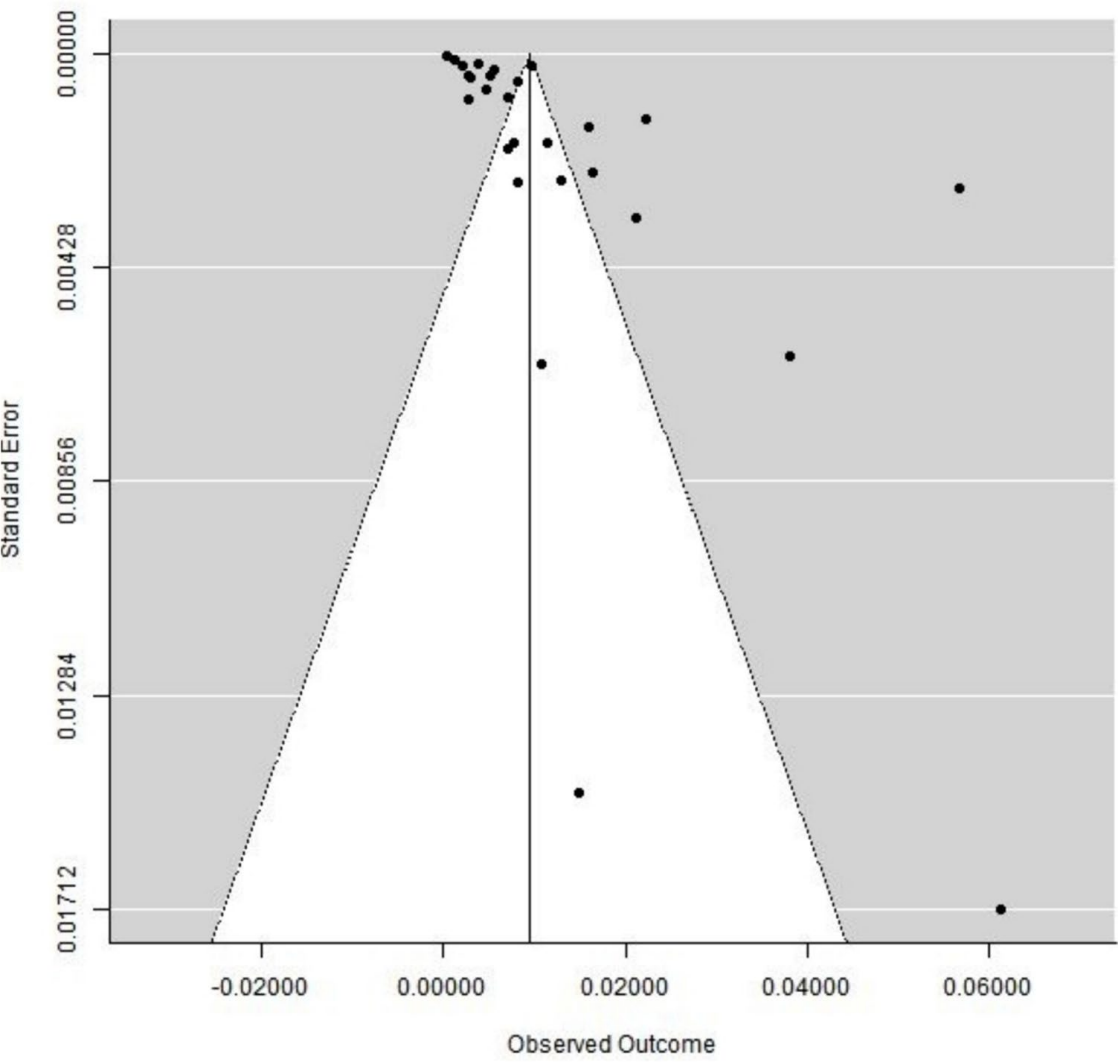
Funnel plot of the included studies

A funnel plot was plotted to look for publication bias present in the included studies, which was asymmetric in nature (Fig. 6). Risk of bias assessment was done by using the NHLBI scale and is shown in Tables 2.

**Table 2:** Risk of bias assessment by using NHLBI quality assessment tool

S. no	Study author and year	NHLBI criteria number													
		1	2	3	4	5	6	7	8	9	10	11	12	13	14
1	Kulkarni ML et al. [12]	Yes	Yes	Yes	Yes	No	No	Yes	NA	Yes	No	Yes	No	NA	No
2	Agarwal SS et al. [13]	Yes	Yes	Yes	Yes	No	No	Yes	NA	Yes	No	Yes	No	NA	No
3	Sood M et al. [14]	Yes	Yes	Yes	Yes	No	No	Yes	NA	Yes	No	Yes	No	NA	No
4	Gupta G et al. [15]	Yes	Yes	Yes	Yes	No	No	Yes	NA	Yes	No	Yes	No	NA	No
5	Gupta RK et al. [16]	Yes	Yes	Yes	Yes	No	No	Yes	NA	Yes	No	Yes	No	NA	No
6	Cherian A et al. [17]	Yes	Yes	Yes	Yes	No	No	Yes	NA	Yes	No	Yes	No	NA	No
7	Mahadevan B et al. [18]	Yes	Yes	Yes	Yes	No	No	Yes	NA	Yes	No	Yes	No	NA	No
8	Shylaja DK et al. [19]	Yes	Yes	Yes	Yes	No	No	Yes	NA	Yes	No	Yes	No	NA	No
9	Kaur G et al. [20]	Yes	Yes	Yes	Yes	No	No	Yes	NA	Yes	No	Yes	No	NA	No
10	Sarkar S et al. [21]	Yes	Yes	Yes	Yes	No	No	Yes	NA	Yes	No	Yes	No	NA	No
11	Babu S et al. [22]	Yes	Yes	Yes	Yes	No	No	Yes	NA	Yes	No	Yes	No	NA	No
12	Jaikrishan G et al. [23]	Yes	Yes	Yes	Yes	No	No	Yes	NA	Yes	No	Yes	No	NA	No
13	Agrawal D et al. [24]	Yes	Yes	Yes	Yes	No	No	Yes	NA	Yes	No	Yes	No	NA	No
14	Sachdeva S et al. [25]	Yes	Yes	Yes	Yes	No	No	Yes	NA	Yes	No	Yes	No	NA	No
15	Kandasamy V et al. [26]	Yes	Yes	Yes	Yes	No	No	Yes	NA	Yes	No	Yes	No	NA	No
16	Bhide P et al. [27]	Yes	Yes	Yes	Yes	No	No	Yes	NA	Yes	No	Yes	No	NA	No
17	Cherian AG et al. [28]	Yes	Yes	Yes	Yes	No	No	Yes	NA	Yes	No	Yes	No	NA	No
18	Pandey U et al. [29]	Yes	Yes	Yes	Yes	No	No	Yes	NA	Yes	No	Yes	No	NA	No
19	Laharwal M et al. [30]	Yes	Yes	Yes	Yes	No	No	Yes	NA	Yes	No	Yes	No	NA	No
20	Rai SK et al. [5]	Yes	Yes	Yes	Yes	No	No	Yes	NA	Yes	No	Yes	No	NA	No
21	Kant S et al. [31]	Yes	Yes	Yes	Yes	No	No	Yes	NA	Yes	No	Yes	No	NA	No
22	Kar A et al. [32]	Yes	Yes	Yes	Yes	No	No	Yes	NA	Yes	No	Yes	No	NA	No
23	Kumar M et al. [4]	Yes	Yes	Yes	Yes	No	No	Yes	NA	Yes	No	Yes	No	NA	No
24	Tiwari P et al. [33]	Yes	Yes	Yes	Yes	No	No	Yes	NA	Yes	No	Yes	No	NA	No
25	Kumar J et al. [34]	Yes	Yes	Yes	Yes	No	No	Yes	NA	Yes	No	Yes	No	NA	No
26	Nagar GG et al. [35]	Yes	Yes	Yes	Yes	No	No	Yes	NA	Yes	No	Yes	No	NA	No
27	Pagolu K et al. [36]	Yes	Yes	Yes	Yes	No	No	Yes	NA	Yes	No	Yes	No	NA	No
NHLBI study quality assessment tool criteria															
Criteria number	Criteria														
1	Was the research question or objective in this paper clearly stated?														
2	Was the study population clearly specified and defined?														
3	Was the participation rate of eligible persons at least 50%?														
4	Were all the subjects selected or recruited from the same or similar populations (including the same time period)? Were inclusion and exclusion criteria for being in the study prespecified and applied uniformly to all participants?														
5	Was a sample size justification, power description, or variance and effect estimates provided?														
6	For the analyses in this paper, were the exposure(s) of interest measured prior to the outcome(s) being measured?														
7	Was the timeframe sufficient so that one could reasonably expect to see an association between exposure and outcome if it existed?														
8	For exposures that can vary in amount or level, did the study examine different levels of the exposure as related to the outcome (e.g., categories of exposure, or exposure measured as continuous variable)?														
9	Were the exposure measures (independent variables) clearly defined, valid, reliable, and implemented consistently across all study participants?														
10	Was the exposure(s) assessed more than once over time?														
11	Were the outcome measures (dependent variables) clearly defined, valid, reliable, and implemented consistently across all study participants?														
12	Were the outcome assessors blinded to the exposure status of participants?														
13	Was loss to follow-up after baseline 20% or less?														
14	Were key potential confounding variables measured and adjusted statistically for their impact on the relationship between exposure(s) and outcome(s)?														

## Discussion

In our meta-analysis, 27 studies were included through which we calculated the overall prevalence rate of NTD in India in our study to be 9.46 per 1000 births. A previous meta-analysis conducted in 2013 by Bhide et al. based on 19 studies reported an overall prevalence of 4.1 per 1000 births. These findings imply that there has been an increased incidence rate of NTDs in India during the last 11 years [7].

The increased prevalence rate of NTDs can be interpreted with a balanced viewpoint focusing on both positive and negative aspects. The positive aspects include improved diagnostics, reporting, changes in the healthcare policy, and more institutional or skilled birth attendant delivery. The negative aspects emphasize the risk of NTDs such as nutritional, environmental, socioeconomic, genetic, and epidemiological factors.

Many government programs launched in India to improve fetal and maternal well-being could have contributed to the increased detection rate of NTDs. The increased number of institutional deliveries has helped in the increased detection. There has been more than a double percentage of increase in institutional deliveries in the year 2019–2021 (88.6%) compared to the year 2005–2006 (40.8%) as per the National Family Health Survey-5 (NFHS-5) [37]. Although the numbers have increased, the low-performing states (LPS) in India, namely, Madhya Pradesh, Uttar Pradesh, Odisha, Rajasthan, Bihar, Chhattisgarh, Jharkhand, and Uttarakhand, still contribute to the significant percentage of home deliveries in India (26%). This explains the regional variation in the number of NTDs observed in the Northern and Eastern regions of India [38].

More emphasis on early detection through antenatal imaging is the need of the hour, which can be achieved by the implementation of consistent screening methods early in the first trimester with the help of trained sonologists [39].

Also, as a part of the Reproductive Maternal Neonatal Child and Adolescent (RMNCH + A) Health Strategy, the Pradhan Mantri Surakshit Matritva Abhiyan (PMSMA) program was launched in the year 2016, which ensures to provide antenatal services including antenatal checkup (ANC) in the second and third trimester at 9th of every month [9]. This program aims to identify high-risk pregnancies and fetal anomalies including NTD through ultrasonographic imaging (USG). This detection continues even after birth through “The Rashtriya Bal Swasthya Karyakram” (RBSK), a program launched in the year 2013, aimed at screening children from birth to 18 years of age for 4D’s: Defects at birth, Deficiencies, Diseases, and Developmental delays. Under this program, a detailed clinical examination is conducted on all babies within 48 h of birth to diagnose birth defects including NTDs [40].

Till the year 2020, the legally permissible limit for medical termination of pregnancy (MTP) in India was 20 weeks. However, the recent amendment to the act in the year 2021 has allowed MTP for congenital birth defects including NTD till 24 weeks by registered medical practitioners (RMPs) and by permission of the medical board beyond 24 weeks of gestation [41]. This can help in decreasing the prevalence in the upcoming years.

The initiative taken by the government’s RMNCH + A strategy necessitates the preconception consumption of folic acid 400 µg for 3 months. This is continued in pregnancy where the dose is increased to 500 µg FA to prevent the development of NTDs [42]. Although the consumption of folic acid decreases the incidence of NTDs, adherence to the intake of folic acid in the preconception period and throughout pregnancy is questionable. Awareness about the importance of folic acid supplementation especially during pregnancy is poor in India which has been reported in different studies. A study done in Raipur showed that women had poor knowledge and awareness regarding the importance of folic acid supplementation [43]. Another study done by Vashisht et al. in 2023 found very low awareness among the mothers of NTD children regarding preconception intake of folic acid, and none of them has consumed it preconceptionally [44]. Addressing these issues via vigilant monitoring or fortification of staples can considerably decrease the prevalence of NTDs. The US Food and Drug Administration in the year 1998 proposed fortification of wheat flour with folic acid to overcome this issue, and many countries have implemented this [45]. A systematic review conducted by Lancellotti et al. in the year 2012 reported a decreased incidence of NTDs in the countries that mandated wheat flour fortification [46]. Considering the large wheat-consuming population in India, it is imperative that the Government of India can take measures to fortify wheat with folic acid to prevent one of the modifiable causes of NTD.

As the lessons learn from the abovementioned studies, patients should be educated and made more aware about the other modifiable risk factors to prevent NTD, such as birth spacing, diabetes, obesity, drug exposure, hyperthermia, and malnutrition. Genetic education and counseling regarding the same to both parents should be done. All these measures can help decrease the incidence of NTD.

Although our study showed the overall and zonal prevalence of NTD, we fail to find the contributing factors of NTDs in each part of the country due to a lack of data.

### Future directions for preventing NTDs in India

Food fortification as previously mentioned can play a key role in decreasing NTDs as it can have a greater reach and is also cost-effective. Additionally, distribution and follow-up of folic acid tablet intake by females at the preconceptual stages for planned pregnancy would also yield better results. Further, some active participation of non-governmental organizations (NGOs) in reaching far-affected places where the incidence of NTDs is significant can bear fruitful results in lowering the occurrence of NTDs in Indian society. Organizing street skits like nukkad natak can have far-reaching effects and can be an eye-opener to many. Spread of the message to the masses through advertising in newspapers and conducting educational seminars at every level, from hospitals to health centers, and at community levels, can enable reaching the information to masses. More studies focusing on the underlying cause, targeting individual regions in India, and considering the diversity of the population can help in reducing the numbers of NTD.

## Conclusion

NTDs are highly debilitating and many a times life-threatening conditions which can be prevented at all primary, secondary, and tertiary levels of prevention. Preconceptional administration of folic acid and screening for genetic diseases can prevent NTDs from occurring in a population. The secondary level of prevention constitutes antenatal radiological screening and management of such cases in utero, if possible, to reduce the severity of the cases. If incompatible with life, pregnancy can be terminated as early as possible to reduce the mental, physical, and social bearing on the pregnant woman and family. Once diagnosed after birth, cases of NTD can be treated surgically and physical rehabilitation can be done thereafter. Also, grass root level workers should be educated about this so that they can encourage early antenatal visits and screening of pregnant women. Hence, we see that with adequate and proactive participation from the government side and policy makers, this problem can be sorted out, and the incidence of NTDs can be brought down.

## Data Availability

No datasets were generated or analyzed during the current study.
